# Theophylline is able to partially revert cachexia in tumour-bearing rats

**DOI:** 10.1186/1743-7075-9-76

**Published:** 2012-08-21

**Authors:** Mireia Olivan, Jochen Springer, Sílvia Busquets, Anika Tschirner, Maite Figueras, Miriam Toledo, Cibely Fontes-Oliveira, Maria Inés Genovese, Paula Ventura da Silva, Angelica Sette, Francisco J López-Soriano, Stefan Anker, Josep M Argilés

**Affiliations:** 1 Cancer Research Group, Departament de Bioquímica i Biologia Molecular, Facultat de Biologia, Universitat de Barcelona, Diagonal 645, Barcelona, 08028, Spain; 2 Institut de Biomedicina de la Universitat de Barcelona, Barcelona, Spain; 3 Division of Applied Cachexia Research, Department of Cardiology, Charité Medical School, Berlin, Germany

**Keywords:** Cachexia, Nutraceuticals, Muscle wasting, Proteolytic system, Heart

## Abstract

**Background and aims:**

The aim of the present investigation was to examine the anti-wasting effects of theophylline (a methylxantine present in tea leaves) on a rat model of cancer cachexia.

**Methods:**

The *in vitro* effects of the nutraceuticals on proteolysis were examined on muscle cell cultures submitted to hyperthermia. Individual muscle weights, muscle gene expression, body composition and cardiac function were measured in rats bearing the Yoshida AH-130 ascites hepatoma, following theophylline treatment.

**Results:**

Theophylline treatment inhibited proteolysis in C2C12 cell line and resulted in an anti-proteolytic effect on muscle tissue (soleus and heart), which was associated with a decrease in circulating TNF-alpha levels and with a decreased proteolytic systems gene expression. Treatment with the nutraceutical also resulted in an improvement in body composition and cardiac function.

**Conclusion:**

Theophylline - alone or in combination with drugs - may be a candidate molecule for the treatment of cancer cachexia.

## Background

The development of cancer cachexia is the most common manifestation of advanced malignant disease. Indeed, cachexia occurs in the majority of terminally ill cancer patients, and it is responsible for the death of 22% of cancer patients [1]. The abnormalities associated with cancer cachexia include anorexia, weight loss, muscle loss and atrophy, anaemia and alterations in carbohydrate, lipid and protein metabolism [2]. Some of these effects are associated also with anti-tumour treatment. The degree of cachexia is inversely correlated with the survival time of the patient and it always implies a poor prognosis [3]. Perhaps one of the most relevant characteristics of cachexia is that of asthenia, which reflects the important muscle wasting that takes place in the cachectic cancer patient [4]. Lean body mass depletion is one of the main trends of cachexia and it involves not only skeletal muscle but it also affects cardiac proteins, resulting in important alterations in heart performance. In addition to the increased muscle protein degradation found during cancer growth, the presence of the tumour also induces an increased rate of DNA fragmentation in skeletal muscle in both rats and mice [5].

One of the factors contributing to wasting during cancer is muscle loss through the activation of proteolysis [6]. The precise mechanism by which intracellular proteins are degraded is not fully understood, although it is accepted that proteolysis may occur inside and outside the lysosomes. The ATP-ubiquitin-dependent proteolytic system has been shown to be involved in the alterations of protein metabolism related to several pathophysiological conditions such as cancer, chronic infection and chronic heart failure [7]. During these pathological conditions commented on, muscle wasting leads to cachexia, a syndrome characterized by weight loss and profound metabolic abnormalities. Unfortunately therapeutic approaches to stop muscle wasting have not been very satisfactory, partly because of the toxicity of inhibitors of the ubiquitin-dependent proteolytic system.

Theophylline (1,3-dimethylxanthine) is an alkaloid that belongs to the same family as caffeine and theobromine [8]. It is present in tealeaves and chocolate and its main effects seem related with relaxation of bronchial muscles together with peripheral blood vessel dilatation [9]. It is for this reason that the alkaloid has been used in the treatment of asthma and patients with chronic obstructive pulmonary disease (COPD) [10,11]. In addition, theophylline decreases circulating TNF-α and increases plasma IL-10, an anti-inflammatory cytokine [12]. Additionally, theophylline is able to increase both the expression and the amount of the gene PPAR-γ, therefore suggesting an additional anti-inflammatory potential [13].

Taking into consideration its anti-inflammatory properties, theophylline could be a good candidate for the treatment of muscle wasting. Bearing this in mind, we have investigated the effects of theophylline treatment both *in vitro*, in an hyperthermia model which we previously reported as a suitable model for studying the anti-proteolytic potential of drugs used in the treatment of muscle wasting [14], and *in vivo*, in rats bearing the Yoshida AH-130 ascites hepatoma, a tumour which induces a high degree of muscle wasting associated with cachexia.

## Methods

### Cell culture

C2C12 mouse skeletal muscle cells were obtained from the American Type Culture Collection. Cells were passaged in high-glucose Dulbecco’s modified Eagle’s medium (DMEM) supplemented with 10% fetal bovine serum (FBS), 100 U/mL penicillin, 100 μg/mL streptomycin, 25 ng/ml fungizone, 110 μg/mL sodium pyruvate, and 2 mM L-glutamine, in a humidified atmosphere of 5% CO_2_ and 95% air at 37°C. For experimental analyses, cells were seeded at 3.7 × 10^4^ cells/cm^2^ in 10% FBS/DMEM until they reached 90–100% confluence 24 h later. At this time, the medium was replaced by DMEM containing 10% horse serum (HS) for induction of differentiation for genetically modified cells. Abundant myotube formation, monitored microscopically, occurred after 4 days in 10% horse serum HS/DMEM. Such fused myotube cultures were utilized for experimental analysis 5 days after transferring the cells to 10% HS/DMEM.

### Hyperthermia model and measurement of protein degradation

C2C12 myotubes were pre-labelled with L-[2,6-^3^ H]phenylalanine (Amersham, Bucks, UK) as described [15] for a 24 h period, after which they were washed extensively in PBS, and incubated in fresh DMEM for a 2 h period at 37°C, until no more radioactivity appeared in the medium. Protein degradation was measured by the release of [2,6-^3^ H]phenylalanine into the medium (DMEM-supplemented with 1% glutamine, 1% penicillin-streptomycin-fungizone and 10%HS) after 6 h incubation at 37 or 41°C according to Gulve *et al*[16]. After the incubation, the cells were rinsed twice in PBS. Cultures were incubated with or without different concentrations of theophylline (0.01 and 0.1 mM) [17,18]. The hyperthermia method to induce protein degradation has been previously described by Smith *et al.*[19].

### Animals

Male Wistar rats (Harlan, Barcelona, Spain) of 5 weeks (initial body weight = 154 g) of age were used in the different experiments. The animals were maintained at 22 ± 2°C with a regular light–dark cycle (light on from 08:00 a.m. to 08:00 p.m.) and had free access to food and water. The food intake was measured daily. All animal manipulations were made in accordance with the European Community guidelines for the use of laboratory animals. Ethical approval was obtained from both the University of Barcelona and the Generalitat de Catalunya Ethics Comittees.

### Tumour inoculation and treatment

Rats were divided into 2 groups namely control and tumour hosts. The tumour rats received an intraperitoneal inoculum of 10^8^ AH-130 Yoshida ascites hepatoma cells obtained from tumours that were in exponential phase of growth as previously described by our group [6,20]. The tumour group was divided into treated and untreated, the former being administered a daily intragastric (i.g.) dose of theophylline (50 mg/kg body weight (bw), dissolved in corn oil) [9] and the latter a corresponding volume of solvent (corn oil). On day 7 after tumour transplantation, the animals, showing a high degree of cachexia accompanied by a marked weight loss [21], were weighed and anaesthetized with an i.p. injection of ketamine/xylazine mixture (3:1) (Imalgene® and Rompun® respectively). The tumour was harvested from the peritoneal cavity and its volume and number of cells evaluated. Tissues were rapidly excised, weighted, and frozen in liquid nitrogen.

### Body composition analysis

A nuclear magnetic resonance spectroscopy device (EchoMRI-700™, Echo Medical Systems, Houston, TX) was used to assess body composition with a sensitivity of 2 g. Fat, muscle, and tissue-free body fluids generate different signals in response to various radio frequency pulses at distinct static magnetic fields [22]. A series of radio pulses at 2 MHz induce changes in magnetic polarization of hydrogen nuclei. These changes decay, or “relax”, with different relaxation times in different substances (http://www.echomri.com). The analyser is sensitive to NMR relaxation times in a range containing those of fat, lean, and saline, and so it detects and differentiates their amounts. In this study, body composition was analysed one day before starting the treatment and one day before sacrifice (7 days), and the results are expressed as the difference between both measurements [23].

### Echocardiographic study

Rats were anesthetized using 1.5% isoflurane and laid in supine position on a platform with all legs taped to ECG electrodes for heart rate monitoring. Body temperature was monitored and maintained at 36–38°C using a heating pad. All hair was removed from the chest. A high resolution echocardiography system (Vevo 770; VisualSonics Inc, Toronto, Canada) was used. The following parameters were assessed using M-mode and: the thickness of intraventricular septum (IVS), Left ventricle diameter (LVD), posterior wall thickness (PWT). The LV end-diastolic volume (LV Vol dia) and end-systolic volume (LV Vol sys) were traced and calculated in B-mode to account for altered ventricular geometry [23]. In this study, echocardiography was performed one day before starting the treatment (results not shown) and one day before sacrifice (7 days).

### RNA isolation

Total RNA from heart and soleus muscle was extracted by TriPureTM kit (Roche, Barcelona, Spain), a commercial modification of the acid guanidinium isothiocyanate/phenol/ chloroform method [24].

### Real-time PCR (polymerase chain reaction)

First-strand cDNA was synthesized from total RNA with oligo dT15 primers and random primers pdN6 by using a cDNA synthesis kit (Transcriptor Reverse Transcriptase, Roche, Barcelona, Spain). Analysis of mRNA levels for the genes from the different proteolytic systems was performed with primers designed to detect the following gene products: ubiquitin (Forward 5^′^ GATCCAGGACAAGGAGGGC 3^′^, Reverse 5^′^ CATCTTCCAGCTGCTTGCCT3′); E2 (Forward 5^′^ AGGCGAAGATGGCGGT 3^′^; Reverse 5^′^ TCATGCCTGTCCACCTTGTA 3^′^); C2 (Forward 5^′^ GTTTCCATTGGGATTGTTGG 3^′^; Reverse 5^′^ TGTTCCATTGGTTCATCAGC 3^′^); C8 proteasome subunit (Forward 5^′^ CAACCATGACAACCTTCGTG 3^′^; Reverse 5^′^ GCCTCAAGC CTTCTCTT TG 3^′^); MuRF-1 (Forward 5^′^ ATCACTCAGGAGCAGGAGGA 3^′^; Reverse 5^′^ CTT GGCACTCAAGAGGAAGG 3^′^); Atrogin-1 (Forward 5^′^ GTTTCCATTGGGATTGTTGG 3^′^; Reverse 5^′^ TGTTCCATTGGTTCATCAGC 3^′^); m-calpain (Forward 5^′^ TTGAGCTGCAGACCATC 3^′^; Reverse 5^′^ GCAGC TTGAAACCTGCTTCT 3^′^) and cathepsin B (Forward 5^′^ CTGCTGAGGACCTGCTTAC 3^′^; Reverse 5^′^ CACAGGGAGGG ATGGTGTA 3^′^); 18 S (Forward 5^′^ CGCAGAATTCCCACTCCCGACCC 3^′^; Reverse 5^′^C CCAAGCTCCAACTA CGAG C 3^′^). To avoid the detection of possible contamination by genomic DNA, primers were designed in different exons. The real-time PCR was performed using a commercial kit (LightCycler TM FastStart DNA MasterPLUS SYBR Green I, Roche, Barcelona, Spain). The relative amount of all mRNA was calculated using comparative CT method. 18 S mRNA was used as the invariant control for all studies.

### Statistical analysis

Statistical analysis of the data was performed by means of one-way and two-way analysis of variance (ANOVA) (post-test Duncan).

## Results and discussion

**Figure 1:**
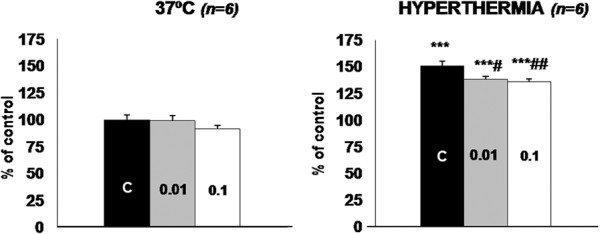
**Effect of theophylline on the proteolytic rate of C2C12 cells under hyperthermia.** Values for protein degradation are presented as the percentage of the respective control value at 37°C of the amino acid radioactivity in the medium versus the total radioactivity incorporated into protein (for further details, see the Material and Methods section). All data are means ± SEM. The number inside the bars represents the concentration of theophylline (mM). Values that are significantly different by one-way ANOVA (post-test Duncan), from the control (non treated) group are indicated by: # *p* < 0.05, ## *p* < 0.01. Values that are significantly different by one-way ANOVA (post-test Duncan) from hyperthermia are indicated by: *** *p* < 0.001.

In order to test the proteolytic modulatory capacity of theophylline (a nutraceutical present in tea), we chose the model of hyperthermia [19]. Indeed, C2C12 cells exposed to 41°C for 6 hours demonstrated a significant increase in proteolytic rate, and theophylline was able to significantly reduce the proteolysis during hyperthermia (0.01 mM 9% and 0.1 mM 10%) (Figure 1).

**Figure 2:**
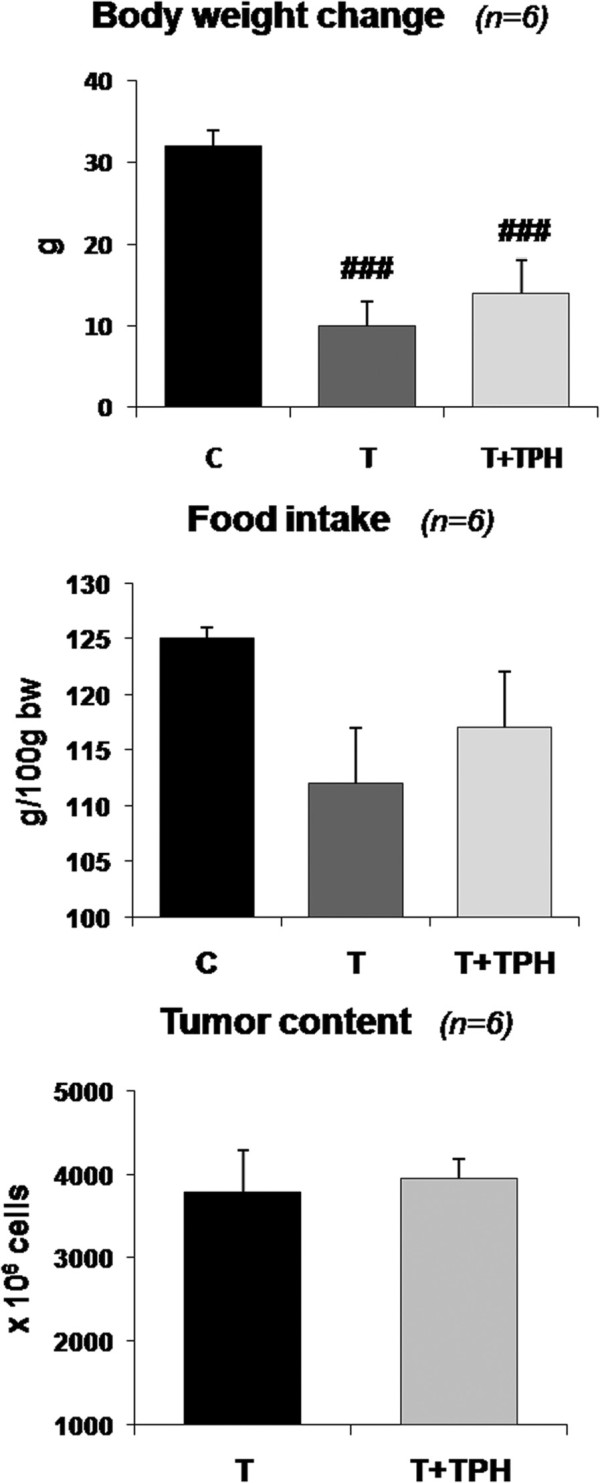
**Effect of theophylline (50 mg/kg bw) on body weight change, food intake and tumour content in rats bearing the Yoshida AH-130 ascites hepatoma.** Results are mean ± SEM. Body weight change is expressed as the difference between final body weight and initial body weight (IBW). Food intake is expressed in g/100 g IBW and refers to the food ingested during the period of the experiment prior to sacrifice, which took place 7 days after tumour inoculation. Tumour cell content is expressed in millions of cells. C = non-tumour-bearing rats, T = tumour-bearing rats, T + TPH = tumour-bearing rats treated with theophylline. Values that are significantly different by two-way analysis of variance (ANOVA) are indicated by: ### *p* < 0.001 (tumour effect).

**Figure 3:**
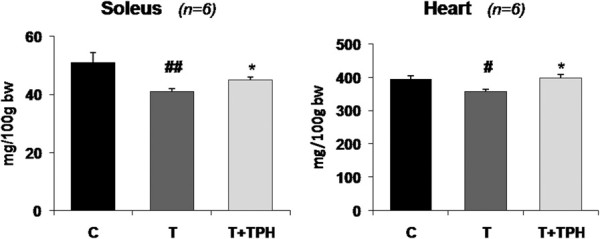
**Effect of theophylline (50 mg/kg bw) on soleus and heart weights in rats bearing the Yoshida AH-130 ascites hepatoma.** Results are mean ± SEM. Muscles weights are expressed as mg/100 g of initial body weight (IBW). C = non-tumour-bearing rats, T = tumour-bearing rats, T + TPH = tumour-bearing rats treated with theophylline. Values that are significantly different by two-way analysis of variance (ANOVA) are indicated by: # *p* < 0.05, ## *p* < 0.01 (tumour effect); * *p* < 0.05 (treatment effect).

Bearing in mind the results obtained *in vitro*, the next aim of the present investigation was to see if this alkaloid was also able to influence muscle proteolysis *in vivo*. We investigated the effects of theophylline treatment in rats bearing the Yoshida AH-130 ascites hepatoma. We have previously shown that the cachectic syndrome is invariably associated with weight loss with skeletal muscle waste in different experimental cancer models [20,25] also observed in the present study (Figures 2 and 3). Theophylline treatment did not influence food intake in the tumour bearing animals, neither did it influence tumour growth (Figure 2). These results are in contrast with previous studies demonstrating that theophylline induces apoptosis and growth inhibition in tumour cells in vitro [26]; also, *in vivo* treatment with theophylline of mice implanted with melanoma cells results in a marked decrease in hepatic and pulmonary metastases [27].

From the results depicted in Figure 2 it can be seen that, in spite of the fact that theophylline treatment resulted in a tendency for higher body weight, the difference did not reach statistical significance. However, the treatment with this alkaloid resulted in a significant increase in the weight of soleus (10%) and cardiac muscles (11.5%), both of them with red and aerobic fibres (Figure 3). Conversely, no effects of treatment were observed with other muscles (non significant differences: *gastrocnemius* T = 572 ± 13 (5) vs T + TPH = 580 ± 12 (6); *EDL* T = 44 ± 1 (5) vs T + TPH = 43.7 ± 1 (6); *tibialis* T = 184 ± 6 (5) vs T + TPH = 185 ± 6 (6)). These results are in agreement with the body composition of the animals. Indeed, as shown in Table 1, a significant decrease of fat and lean mass was observed due to the tumour growth. The treatment with theophylline resulted in a tendency for higher lean body mass, although the difference did not reach statistical significance. Interestingly, however, the animals treated with theophylline lost significantly less fat mass (Table 1). This observation is very surprising since theophylline has been demonstrated to be a lipolytic agent both in experimental animals [28] and humans [29], its mechanism of action being based on the inhibition of cAMP phosphodiesterase (PDE) [30]. These data suggest that an abnormal regulation of adipose tissue metabolism associated with tumour burden exists. The inhibition of PDE could be the mechanism underlying the effects of theophylline on muscle tissue, in this case on an aerobic muscle, this idea has been already suggested by others while studying muscle protein catabolism in septic rats [31].

**Table 1:** Effects of theophylline (50 mg/kg bw) on body composition in rats bearing the Yoshida AH-130 ascites hepatoma

		***Experimental group***			
	**C*(n = 6)***	**T*(n = 10)***	**T + TPH*(n = 7)***	**ANOVA**	
				**A**	**B**
**Fat mass (g)**	3.2 ± 1.1	−4.3 ± 0.5	−2.3 ± 0.7	0,000	0,045
**Lean body mass (g)**	23.5 ± 1.5	1.7 ± 1.7	7 ± 3	0,000	ns
**Fluids (g)**	0.03 ± 0.2	−0.7 ± 0.2	0.04 ± 0.3	0,034	0,025

Results are expressed as the difference between day 0 (tumour inoculation) and day 7; mean ± SEM. C = non-tumour-bearing rats, T = tumour-bearing rats, T + TPH = tumour-bearing rats treated with theophylline. Statistical significance of the results by two-way analysis of variance (ANOVA); ns: non-significant differences. A (tumour effect); B (treatment effect).

Muscle catabolism associated with cancer is influenced by an activation of the ubiquitin-dependent proteolytic system [6]. Bearing this in mind, we decided to investigate the effects of theophylline on the gene expression of the different proteolytic systems. The results, presented in Table 2, show an increased expression of some genes involved in the ATP-ubiquitin-dependent proteolytic pathway in heart (ubiquitin, C2, C8, E2, MuRF-1 and Atrogin-1) and soleus muscle (ubiquitin, E2, Atrogin-1 and MuRF-1) of tumor-bearing animals in relation with the corresponding control animals. Tumour-bearing theophylline-treated animals showed a decrease in the expression of ubiquitin (42%) and MuRF-1 (53%), an ubiquitin-dependent ligase that is muscle-specific, in soleus muscle. Theophylline caused a significant decrease in ubiquitin (41%), proteasome subunits C2 (37%), C8 (38%) and E2 (49%) in heart. A tendency to reduce MuRF-1 mRNA content was also observed with theophylline treatment, although the differences did not reach statistical significance.

**Table 2:** Effects of theophylline (50 mg/kg bw) on soleus muscle and heart mRNA content of the different proteolytic systems in rats bearing the Yoshida AH-130 ascites hepatoma

		**SOLEUS**					**HEART**			
**Treatment**	**C*(n = 5)***	**T*(n = 5)***	**T + TPH*(n = 6)***	**ANOVA**		**C*(n = 5)***	**T*(n = 5)***	**T + TPH*(n = 6)***	**ANOVA**	
				**A**	**B**				**A**	**B**
**PROTEOLYTIC SYSTEM**										
*Ubiquitin-dependent*										
Ubiquitin	100 ± 6	204 ± 15	118 ± 18	0,000	0,001	100 ± 5	302 ± 51	177 ± 32	0,001	0,019
Proteasome subunit C2	100 ± 13	168 ± 10	160 ± 40	ns	ns	100 ± 3	303 ± 47	191 ± 23	0,000	0,010
Proteasome subunit C8	100 ± 5	111 ± 8	97 ± 25	ns	ns	100 ± 3	262 ± 26	164 ± 22	0,000	0,005
E2	100 ± 5	527 ± 129	288 ± 80	0,004	0,063	100 ± 5	224 ± 55	114 ± 19	0,013	0,025
MuRF-1	100 ± 3	171 ± 19	80 ± 26	0,025	0,007	100 ± 3	309 ± 92	177 ± 38	0,015	ns
Atrogin-1	100 ± 8	392 ± 22	362 ± 71	0,002	ns	100 ± 3	161 ± 21	152 ± 21	0,039	ns
*Calcium-dependent*										
m-calpain	100 ± 4	117 ± 23	142 ± 43	ns	ns	100 ± 4	307 ± 58	170 ± 18	0,001	0,009
*Lysosomal*										
Cathepsin-B	100 ± 4	111 ± 8	151 ± 34	ns	ns	100 ± 2	194 ± 40	111 ± 13	0,010	ns

For further details, see the Material and Methods section. The results are expressed as a percentage of controls (T). All data are mean ± SEM. C = non-tumour-bearing rats, T = tumour-bearing rats, T + TPH = tumour-bearing rats treated with theophylline. Statistical significance of the results by two-way analysis of variance (ANOVA) are indicated by: ns (non-significant differences), A (tumour effect), B (treatment effect).

Concerning other proteolytic systems, tumor burden also resulted in an increase in the expression of the calcium-dependent system *m- calpain* and in the lysosomal *cathepsin* B in heart (Table 2)*.* This proteolytic mechanism has also been shown to play a role in cancer cachexia [32]. Theophylline, in the heart, caused a significant decrease in m-calpain (45%) mRNA, a component the calcium-dependent proteolytic system.

**Figure 4:**
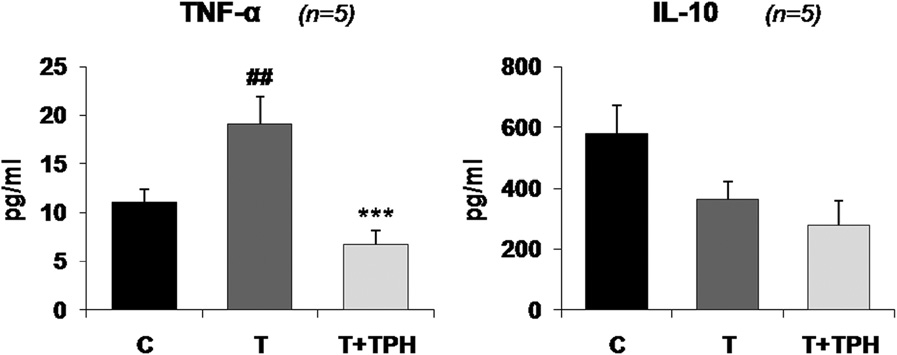
**Effect of theophylline (50 mg/kg bw) on circulating TNF-α and IL-10 concentrations in rats bearing the Yoshida AH-130 ascites hepatoma.** Results are mean ± SEM. The plasmatic concentrations of TNF-α and IL-10 are expressed as pg/ml of plasma. C = non-tumour-bearing rats, T = tumour-bearing rats, T + TPH = tumour-bearing rats treated with theophylline. Values that are significantly different by two-way analysis of variance (ANOVA) are indicated by: ## *p* < 0.01 (tumour effect); *** *p* < 0.001 (treatment effect).

As previously stated, theophylline is able to have anti-inflammatory effects [13]. Therefore, we examined the effects of the alkaloid on plasma cytokine levels. The results, depicted in Figure 4, show that the treatment significantly decreases TNF-α, (significantly increased due to the presence of the tumour when compared with control animals), while it had no effects on IL-10 (an anti-inflammatory cytokine). Since TNF-α has been shown to influence skeletal muscle proteolysis during cancer cachexia [33], one may speculate that the effects of theophylline on muscle weight and proteolysis could be accounted for the changes in this pro-cachectic cytokine [4].

Based on a clinical study with more than 4000 autopsy reports, Houten and Reilley [34] suggested that heart problems are responsible for at least 11% of cancer deaths. In fact, these data could be underestimated since a high percentage of deaths are actually attributed to infections, drug-induced toxicity and alterations in the osmotic balance, which are mainly due to heart problems. Therefore, more than 20% of the mortality is due to alterations in heart function. Indeed, McBride and collaborators [35] observed that more than 50% of multiple myeloma patients suffered cardiac failure during the neoplastic process. Data from our own laboratory also indicate that tumours implanted in experimental animals resulted in a decrease of the heart weight [20]. Drott and Lundholm [36] observed an increase in oxygen consumption associated with the heart in an experimental model of cancer. Important ultrastructural changes characterized by an increase in the ratio of myofibrilles/mitochondria and sarcomeric alterations in a similar way as observed during cardiac failure were also reported. Taking this into consideration, and also the observed effects of theophylline on cardiac mass, we decided to investigate heart parameters in the experimental rat model used in our study. The results presented in Table 3 show that implantation of the tumour resulted in a posterior wall thickness (PWT) size decrease. This was associated with a decreased left ventricle mass (LV). Interestingly, treatment with theophylline increases these parameters, normalizing their values (Table 3).

**Table 3:** Effects of theophylline (50 mg/kg bw) on heart parameters in rats bearing the Yoshida AH-130 ascites hepatoma

		***Experimental group***			
	**C(n = 5)**	**T(n = 8)**	**T + TPH(n = 9)**	**ANOVA**	
				**A**	**B**
**LV ejection fraction (%)**	77 ± 2	73 ± 2	72 ± 3	ns	ns
**Fractional shortening (%)**	26 ± 6	23 ± 1	20 ± 3	ns	ns
**LVD dia (mm)**	12 ± 0.1	2 ± 0.3	11 ± 0.2	ns	ns
**LVD sys (mm)**	9 ± 0.7	9 ± 0.2	10 ± 0.4	ns	ns
**PWT dia (mm)**	2.6 ± 0.1	2.4 ± 0.1	3 ± 0.1	ns	0,012
**PWT sys (mm)**	4 ± 0.2	3.2 ± 0.2	3.3 ± 0.2	0.05	ns
**LV Vol dia (μl)**	217 ± 22	220 ± 14	190 ± 17	ns	ns
**LV Vol sys (μl)**	50 ± 8	55 ± 4	48 ± 7	ns	ns
**LVSV (μl)**	167 ± 15	159 ± 10	139 ± 14	ns	ns
**LVmass (mg)**	313 ± 6	274 ± 10	322 ± 14	0,032	0,005

Echocardiographic data of non-tumour bearing rats (C), tumour bearing rats (T) and tumour bearing rats treated with theophylline (T + TPH). Results are mean ± SEM. LV ejection fraction: (LV vol dia-LV vol sys)/LV vol dia; fractional shortening: (LVD dia-LVD sys)/LVD sys; Left ventricle diameter in diastole: LVD dia; Left ventricle diameter in systole: LVD sys; posterior wall thickness in diastole: PWT dia; posterior wall thickness in systole: PWT sys; left ventricle volume in diastole: LV Vol dia; left ventricle volume in systole: LV Vol sys; left ventricle stroke volume: LVSV (LV Vol dia- LV Vol sys); left ventricle mass: LV mass (expressed as mg/100 g of initial body weight). Statistical significance of the results by two-way analysis of variance (ANOVA); ns: non-significant differences. A (tumour effect); B (treatment effect).

## Conclusions

From the results presented here, a potential role of theophylline in the treatment of the muscle wasting associated with cancer – particularly cardiac muscle – is postulated. Unfortunately, there is not a perfect therapeutic strategy for the treatment of muscle wasting associated with cancer. Although a plethora of treatments have been proposed, the use of a single drug does not seem to be an ideal approach [37]. It has become increasingly clear that a multifactorial approach is probably the most suitable treatment. Indeed, combinations of different nutraceuticals with high protein nutrition have been highly successful [38]. Theophylline could perhaps be used in combination with other nutraceuticals, nutrition or drugs in a new approach for the treatment of muscle wasting. Further work is therefore needed to evaluate the anti-wasting effects of theophylline in combination with other treatments.

## Competing interests

All authors of this research have not conflict of interest related with employment, consultancies, stock ownership, honoraria, paid expert testimony, patent applications/ registrations, and grants or other funding.

## Authors’ contributions

Each author has participated sufficiently, intellectually or practically, in the work to take public responsibility for the content of the article, including the conception, design, and conduct of the experiment and for data interpretation (authorship). MO carried out the studies, sample analysis, and data analyses, performed the statistical analysis and helped to draft the manuscript. SB participated in the design and coordination of the study, carried out the studies, and helped to draft the manuscript. JS and MF participated in the design of the study and carried out the studies. AH, MT, CFO, MIG, PV and AS helped to carry out the studies. FJL-S, SA and JMA conceived the study, participated in the design, coordination of the study, drafted the manuscript and revised it critically. All authors have read and approved the final manuscript.
